# Chicken manure application alters microbial community structure and the distribution of antibiotic-resistance genes in rhizosphere soil of *Cinnamomum camphora* forests

**DOI:** 10.1093/femsec/fiad155

**Published:** 2023-11-24

**Authors:** Deqiang Chen, Jiawei Zou, Dexing Chen, Xin He, Cuili Zhang, Jinwei Li, Siren Lan, Zhong-Jian Liu, Shuangquan Zou, Xin Qian

**Affiliations:** Fujian Colleges and Universities Engineering Research Institute of Conservation and Utilization of Natural Bioresources, College of Forestry, Fujian Agriculture and Forestry University, No. 15 Shangxiadian Road, Cangshan District, Fuzhou 350002, Fujian Province, China; Key Laboratory of National Forestry and Grassland Administration for Orchid Conservation and Utilization at Colleage of Landscape Architecture, Fujian Agriculture and Forestry University, No. 15 Shangxiadian Road, Cangshan District, Fuzhou 350002, Fujian Province, China; School of Pharmacy, Fujian Medical University, No. 1 Xuefu North Road, University Town, Fuzhou 350002, Fujian Province, China; Fujian Colleges and Universities Engineering Research Institute of Conservation and Utilization of Natural Bioresources, College of Forestry, Fujian Agriculture and Forestry University, No. 15 Shangxiadian Road, Cangshan District, Fuzhou 350002, Fujian Province, China; Fujian Colleges and Universities Engineering Research Institute of Conservation and Utilization of Natural Bioresources, College of Forestry, Fujian Agriculture and Forestry University, No. 15 Shangxiadian Road, Cangshan District, Fuzhou 350002, Fujian Province, China; Key Laboratory of National Forestry and Grassland Administration for Orchid Conservation and Utilization at Colleage of Landscape Architecture, Fujian Agriculture and Forestry University, No. 15 Shangxiadian Road, Cangshan District, Fuzhou 350002, Fujian Province, China; Key Laboratory of National Forestry and Grassland Administration for Orchid Conservation and Utilization at Colleage of Landscape Architecture, Fujian Agriculture and Forestry University, No. 15 Shangxiadian Road, Cangshan District, Fuzhou 350002, Fujian Province, China; Key Laboratory of National Forestry and Grassland Administration for Orchid Conservation and Utilization at Colleage of Landscape Architecture, Fujian Agriculture and Forestry University, No. 15 Shangxiadian Road, Cangshan District, Fuzhou 350002, Fujian Province, China; Fujian Colleges and Universities Engineering Research Institute of Conservation and Utilization of Natural Bioresources, College of Forestry, Fujian Agriculture and Forestry University, No. 15 Shangxiadian Road, Cangshan District, Fuzhou 350002, Fujian Province, China; Key Laboratory of National Forestry and Grassland Administration for Orchid Conservation and Utilization at Colleage of Landscape Architecture, Fujian Agriculture and Forestry University, No. 15 Shangxiadian Road, Cangshan District, Fuzhou 350002, Fujian Province, China; Fujian Colleges and Universities Engineering Research Institute of Conservation and Utilization of Natural Bioresources, College of Forestry, Fujian Agriculture and Forestry University, No. 15 Shangxiadian Road, Cangshan District, Fuzhou 350002, Fujian Province, China; Key Laboratory of National Forestry and Grassland Administration for Orchid Conservation and Utilization at Colleage of Landscape Architecture, Fujian Agriculture and Forestry University, No. 15 Shangxiadian Road, Cangshan District, Fuzhou 350002, Fujian Province, China; Fujian Colleges and Universities Engineering Research Institute of Conservation and Utilization of Natural Bioresources, College of Forestry, Fujian Agriculture and Forestry University, No. 15 Shangxiadian Road, Cangshan District, Fuzhou 350002, Fujian Province, China; Key Laboratory of National Forestry and Grassland Administration for Orchid Conservation and Utilization at Colleage of Landscape Architecture, Fujian Agriculture and Forestry University, No. 15 Shangxiadian Road, Cangshan District, Fuzhou 350002, Fujian Province, China; College of Life Sciences, Fujian Agriculture and Forestry University, No. 15 Shangxiadian Road, Cangshan District, Fuzhou 350002, Fujian Province, China

**Keywords:** antibiotic-resistance genes, bacterial community, chicken manure organic fertilizer, *Cinnamomum camphora*, environmental factor

## Abstract

The distribution of antibiotic-resistance genes (ARGs) in environmental soil is greatly affected by livestock and poultry manure fertilization, the application of manure will lead to antibiotic residues and ARGs pollution, and increase the risk of environmental pollution and human health. *Cinnamomum camphora* is an economically significant tree species in Fujian Province, China. Here, through high-throughput sequencing analysis, significant differences in the composition of the bacterial community and ARGs were observed between fertilized and unfertilized rhizosphere soil. The application of chicken manure organic fertilizer significantly increased the relative abundance and alpha diversity of the bacterial community and ARGs. The content of organic matter, soluble organic nitrogen, available phosphorus, nitrate reductase, hydroxylamine reductase, urease, acid protease, β-glucosidase, oxytetracycline, and tetracycline in the soil of *C. camphora* forests have significant effects on bacterial community and ARGs. Significant correlations between environmental factors, bacterial communities, and ARGs were observed in the rhizosphere soil of *C. camphora* forests according to Mantel tests. Overall, the findings of this study revealed that chicken manure organic fertilizer application has a significant effect on the bacterial community and ARGs in the rhizosphere soil of *C. camphora* forests, and several environmental factors that affect the bacterial community and ARGs were identified.

## Teaser

The results of this study provide new insights that will aid the development of manure application strategies as well as the sustainable management of *Cinnamonum camphora* forests.

## Introduction

Soil is the largest reservoir of antibiotic-resistance genes (ARGs) in the environment (Allen et al. 2010, Nesme and Simonet 2015), and ARGs in the soil are a major component of the environmental resistome, which plays a major role in determining the resistance profiles of human pathogens (Forsberg et al. 2012, Nadeem et al. 2020). Many ARGs in the environment are derived from livestock manure (McKinney et al. 2018); several ARGs have been identified in chicken, cattle, and pig manure (Wang et al. 2019). Most animal manure is applied to soil as fertilizer, and this mediates the spread of ARGs in the soil (Knapp et al. 2011, Qiao et al. 2018). The antibiotics in livestock manure will also enter the environment and remain for a long time (Yang and Carlson 2003). Although the residual concentration of antibiotics is very low, it will affect the antibiotic resistance. Antibiotic-resistant bacteria induced by antibiotic residues in animal feces are likely to enter the rhizosphere and spread in plants, and then enter the human body through the food chain, which will pose a great threat to human health (Zhang et al. 2012). There is, thus a need to study the accumulation and dissemination of ARGs in manure-treated soil, as such studies can aid the development of strategies to mitigate the human health risks of ARG dissemination.

Although the application of manure can increase the yield of crops in various soil ecosystems, it can introduce large amounts of ARGs to the soil that eventually reach the leaves of plants (Yang et al. 2018, Chen et al. 2018a). Thus, the plant microbiome is the main route by which humans are exposed to ARGs in the environment. An increasing number of studies of environmental ARGs have been conducted, and the human health threats posed by the ARGs carried by plant microbiomes have received increased research attention (Abbassi et al. 2022). ARGs in the microbiome of plants increase via the absorption of antibiotics from agricultural soil; ARGs carried by microorganisms can also enter the endophytic or epiphytic microbiome of plants via the soil and plant roots (Blau et al. 2018). However, the ability of ARGs to spread in plants and soil and its driving factors need to be requires further examination.

The accumulation of antibiotics in soil can impose strong selection for the evolution of resistance to antibiotics in microorganisms, and this can increase the diversity and abundance of ARGs in the soil (Heuer et al. 2011, Shawver et al. 2021). A meta-analysis conducted by Duarte et al. (2019) revealed a significant relationship between the abundance of ARGs and residual antibiotics, even at low antibiotic concentrations. Zhao et al. (2019) found that the content of oxytetracycline (OTC) in greenhouse soil under long-term cow manure and chicken manure application was significantly positively correlated with the abundance of tetracycline ARGs (*tetO, tetW*, and *tetM*). The accumulation of antibiotics in the soil might favor the evolution of antibiotic resistance in soil microorganisms, which could accelerate the spread of ARGs; the application of manure can provide sufficient carbon, nitrogen, and phosphorus for microorganisms in the soil; these alterations in the content of soil nutrients can promote the growth and reproduction of bacteria carrying ARGs as well as horizontal gene transfer (Peng et al. 2016). Changes in the structure of soil bacterial communities induced by long-term fertilization are thought to be the main factor affecting the abundance of ARGs (Forsberg et al. 2014, Chen et al. 2016, Han et al. 2018). Changes in the structure of soil bacterial communities induced by manure application explained 39.8% of the variation in ARGs; the second most important variable was soil nutrient conditions (Chen et al. 2016). Wang et al. (2020) found that the application of manure significantly changed the soil microbial community, which was considered as the main driving factor for the formation of soil ARG profile. Wang et al. (2018) found that long-term application of sewage sludge and chicken manure changed the composition of bacterial community in the phyllosphere, significantly reduced the bacterial alpha diversity, and significantly increased the abundance of ARG. Variation distribution analysis and network analysis showed that ARG spectrum was closely related to bacterial community composition. These results have improved people’s understanding of the diversity of plant-related antibiotic resistance and the factors affecting the distribution of ARG in the phyllosphere; in addition, soil and other environmental characteristics such as soil texture, organic matter, total nitrogen (TN), and soil enzyme activity affect the suitability of the soil environment for microorganisms and, thus the diversity and abundance of ARGs (Chen et al. 2016, Zhou et al. 2017, Yan et al. 2020, Li et al. 2021).

*Cinnamomum camphora* is a member of the family Lauraceae that occurs in tropical and subtropical regions of Asia. It is an economically important tree species in Fujian Province, China, for its high ornamental, medicinal, and economic value (Singh and Jawaid 2012, Chen et al. 2021). Most studies of *C. camphora* forests have analyzed its physiology, essential oil composition, and related secondary metabolites; however, few studies have examined the soil microorganisms in *C. camphora* forests. Recent studies have revealed significant differences in the soil and root-associated bacterial and fungal communities, as well as the diversity of *C. camphora* forests on different slopes (Chen et al. 2022a, b). It is mentioned above that after applying chicken manure fertilizer, more antibiotics and arginine may accumulate in plant soil, which will pollute the surrounding environment (Wang et al. 2019, Zhao et al. 2019). Nonetheless, we still lack a comprehensive understanding of the effects of chicken manure fertilization on the growth of *C. camphora* forests and the diffusion of ARGs in the soil (Chen et al. 2022a, b). Thus, it is of great significance to evaluate the effect of applying chicken manure organic fertilizer on soil-related ARGs and its growth in *C. camphora* forests.

Here, we analyzed the effects of fertilizer application on the structure of soil microbial communities and the distribution of ARGs using 16S rRNA and metagenomic sequencing. We also analyzed soil physicochemical properties, soil enzyme activities, and the content of OTC and tetracycline (Tet) antibiotics in *C. camphora* forests. The aim of this study was to characterize changes in the composition, abundance, and diversity of the rhizosphere soil bacterial community and ARGs of *C. camphora* forests following the application of chicken manure organic fertilizer. We also analyzed the effects of various environmental factors on the soil bacterial community and ARGs in *C. camphora* forests. The results of this study provide new insights that will aid the development of manure application strategies as well as the sustainable management of *C. camphora* forests. Our findings also have implications for the development of strategies to limit the abundances of ARGs in plants and soil.

## Material and methods

### Study site and sample collection

Our study was focused on the *C. camphora* forests in Nan’an, Fujian Province, China (25°16′N, 118°31′E, 800 m). Two adjacent experimental plots were established with an interval of about 150 m, and pig manure fertilizer and organic chemical fertilizer were applied to each plot before 2010; the experimental plots were not subsequently treated with fertilizer for the next 10 years. In one sample plot, chicken manure organic fertilizer was applied twice a year for 2 years starting in 2020, with pH 8.8, organic matter (OM) 49%, TN 2.17%, available phosphorus (AP) 5.70%, and available potassium 4.26%, and still no fertilizer was applied to the other sample plot. The region has a subtropical monsoon climate, with hot and rainy climate in summer and mild and humid climate in winter. The average annual temperature was 19.5°C, and the average annual rainfall was between 800 and 1900 mm.

For each experimental plot, three subplots (10 m × 10 m) were established, all separated by a minimum distance of 10 m. Measurements of the ground diameter, height, crown width, and total biomass were taken from 20 *C. camphora* trees in each subplot (Table S1, Supporting Information).

*Cinnamomum camphora* forests were sampled in mid-December 2022. The five-point and quartering methods were utilized to take soil samples from the 0–20 cm layer, which were then blended into a single sample for each subplot. Soil samples were then sealed and taken to the laboratory for analyses of soil physical and chemical properties, soil enzyme activity, and the content of OTC and Tet antibiotics. Measurements of several soil physical and chemical properties were taken, including pH, electrical conductivity (EC), soil organic matter (SOM), soil soluble organic nitrogen (SON), AP, total carbon (TC), TN, and soil moisture content (SMC). Measurements of soil enzyme activity were taken for the following enzymes: soil nitrite reductase (S-NiR), soil nitrate reductase (S-NR), soil hydroxylamine reductase (S-HR), soil urease (S-UE), soil acid phosphatase (S-ACP), soil peroxidase (S-POD), soil acid protease (S-ACPT), soil dehydrogenase (S-DHA), soil-α-glucosidase (S-α-GC), and soil-β-glucosidase (S-β-GC) (Tables S2 and S3, Supporting Information).

Three trees were randomly selected from each subplot. A sterilized shovel was used to dig the soil of 0–20 cm underground, located about 10 cm away from the root system of *C. camphora*. After sieving through a 2-mm sieve, samples from the same subplot were combined together and placed in a 50-ml sterile centrifuge tube, which was labeled as bulk soil. Samples of fine roots were gathered from each plant by tracing the large roots from the base of the trunk in three directions, acquiring 9–15 root segments that measure 5–8 cm from each subplot.

Phosphate buffer was used to elute the rhizosphere soil attached to the roots (Xiao et al. 2017). First, 25 ml of phosphate buffer was poured into a 50-ml sterile centrifuge tube; it was then placed in a tube along with the roots and vortexed for 15 s at maximum speed on a vortex shaker (Vortex-Genie® 2, Mobio Laboratories Inc., USA). The vortexed suspension was filtered through a 100-μm sterile nylon mesh into a new centrifuge tube and centrifuged for 15 min (3200 × *g*). The resulting precipitate was labeled as the rhizosphere soil. The washed roots were then surface disinfected by submerging them in 0.5% (v/v) sodium hypochlorite solution and gently shaking for 3 min. Following two rinses with sterile water, root samples were placed on sterilized filter paper for drying. All the bulk soil, rhizosphere soil, and root samples were then stored at ‒80°C until the DNA extraction process.

### DNA extraction, 16S rRNA gene sequencing, and sequence analysis

The FastDNA® Spin Kit for Soil (MP Biomedicals, USA) was used to extract DNA from all samples. A NanoDrop 2000 ultraviolet spectrophotometer (Invitrogen, Thermo Fisher Scientific, Waltham, MA, USA) was used to measure the purity of DNA.

The 16S rRNA V3 + V4 variable regions of all DNA samples were amplified using the following primers: 338F (5′-ACTCCTACGGGAGGCAGCA-3′) and 806R (5′-GGACTACHVGGGTWTCTAAT-3′) (Xu et al. 2016). PCR was conducted in 20 µl reactions containing the following components: 10 ng of genomic DNA, 4 µl of 5 × Fast Pfu buffer 4 μl, 2 µl of dNTP (2.5 mM each), 0.8 µl of upstream and downstream primers, 0.4 µl of Fast Pfu DNA polymerase, 0.2 µl of bovine serum albumin, and the rest with double-distilled water. The thermal cycling conditions were as follows: 95°C for 3 min, 30 cycles of 95°C for 30 s, 50°C for 30 s, and 72°C for 45 s; and 72°C for 7 min. An AxyPrep DNA Gel Extraction Kit was used to purify the PCR products, and a miniature fluorometer Quantus™ Fluorometer (Promega, USA) was used to purify the PCR products. Library construction was performed using the NEXTFLEX® Rapid DNA-seq Kit (BiooScientific, USA), and sequencing was conducted using the Illumina NovaSeq 6000 platform.

Quality control of the original sequence data was conducted using FASTP (Magoč and Salzberg 2011) (https://github.com/OpenGene/fastp, version 0.20.0) software, and merge the paired-end reads using Flash. (Chen et al. 2018b) (http://www.cbcb.umd.edu/software/flash, version 1.2.7) software. Sequences were clustered into operational taxonomic units (OTUs) at 97% similarity using UPARSE software (Edgar 2013, Stackebrandt and Goebel 1994) (http://drive5.com/uparse/, version 7.1). To minimize the effect of sequencing depth on the data analysis, the number of sequences of all samples was standardized to 17 898. OTU sequences were aligned against the SILVA database (version 138) using the RDP classifier (70% confidence interval) (Wang 2007) (http://rdp.cme.msu.edu/, version 2.11).

### Metagenomic sequencing and bioinformatics analysis

Metagenomic sequencing of six rhizosphere soil samples was conducted using the Illumina NovaSeq 6000 sequencing platform. The 3′ and 5′ adapter sequences of the reads, low-mass sequences with lengths less than 50 bp, sequences with average base mass values less than 20, and sequences with N bases were removed using FASTP software (Chen et al. 2018b). Optimized sequences were assembled using MEGAHIT software (Li et al. 2015b) (https://github.com/voutcn/megahit, version 1.1.2). Subsequent analysis was conducted on contigs with a length greater than or equal to 300 bp. The open reading frames of contigs were predicted using Prodigal software (Hyatt et al. 2010). Genes with lengths greater than or equal to 100 bp were translated into amino acid sequences. CD-HIT (Fu et al. 2012) (http://www.bioinformatics.org/cd-hit/, version 4.6.1) was used to construct a nonredundant gene set. The original sequences obtained by 16S rRNA and metagenome sequencing have been uploaded to the National Center for Biotechnology Information Sequence Read Archive database under the BioProject numbers PRJNA979820 and PRJNA980165, respectively.

### Analysis of ARGs, Cluster of Orthologous Groups of proteins, and Kyoto Encyclopedia of Genes and Genomes

The amino acid sequences of the nonredundant gene set were compared against the Comprehensive Antibiotic Resistance Database (CARD), as well as the Evolutionary Genealogy of Genes: Non-supervised Orthologous Groups (eggNOG) and Kyoto Encyclopedia of Genes and Genomes (KEGG) databases (BLASTP e-value ≤ 1 × 10^−5^, similarity ≥ 90%, comparison length ≥ 25 bp) using DIAMOND software (Buchfink et al. 2015, 2021) (http://www.diamondsearch.org/index.php, version 2.0.13), and Cluster of Orthologous Groups (COGs) of proteins and KEGG (pathway level) annotation information was obtained for ARGs. Abundances of genes in all rhizosphere soil samples were determined according to the sum of ARGs, as well as the COG and KEGG (pathway level) gene abundances. Functional annotation information was used to identify Tet ARGs in drug class level.

### Statistical analyses

R software version 4.3.0 software was used to conduct all statistical analyses. Principal coordinate analysis (PCoA) based on the Bray–Curtis distance was used to evaluate the effects of fertilization on the bacterial community and functional gene composition, and the vegan package (Oksanen et al. 2007) was used to conduct permutation multivariate analysis of variance (PERMANOVA) to test the significance of the differences. The PMCMR package (Pohlert and Pohlert 2018) was used to calculate the Shannon index of bacteria in different samples, to study the differences in bacterial diversity among different samples, and Wilcoxon rank-sum tests were conducted to characterize intergroup differences in alpha diversity. The stats R package (Keenan et al. 2013) was used to analyze the relative abundances of intergroup differences in species and ARGs between fertilized and unfertilized samples; the threshold for statistical significance was *P* < .05. The Edger package (Robinson et al. 2010) was used to analyze the differential expression patterns of highly differentiated OTUs and functional genes. The Linket package (Zhu et al. 2022) was used to conduct Mantel tests, correlation analysis, and heat map analysis, combined with redundancy analysis (RDA) based on Bray–Curtis distance to determine the relationship between environmental factors and bacterial communities and ARGs. Co-occurrence networks based on the Spearman correlation matrix were constructed using the WGCNA package (Langfelder and Horvath 2008). The nodes in these networks correspond to bacterial OTUs, and the edges indicate the correlations between them. A false discovery rate (FDR) correction for multiple comparisons was used to evaluate the significance of the nodes and edges in the network. The igraph package (Csardi and Nepusz 2006) was used to calculate the network topological features of the bacterial community between fertilized and unfertilized samples (number of nodes, number of edges, degree, clustering coefficient, modularity, average path length, betweenness centrality, and diameter). The relative importance of different environmental factors (soil physicochemical properties, soil enzyme activity, OTC, and Tet) in determining the network topological features was determined using multiple regression on distance matrices (MRM). Correlations between network properties and environmental factors were determined using the “corrplot” and “Hmisc” packages (Harrell and Harrell 2019, Pesenti et al. 2019).

## Results

### Diversity of bacterial communities and ARGs

**Figure 1.**
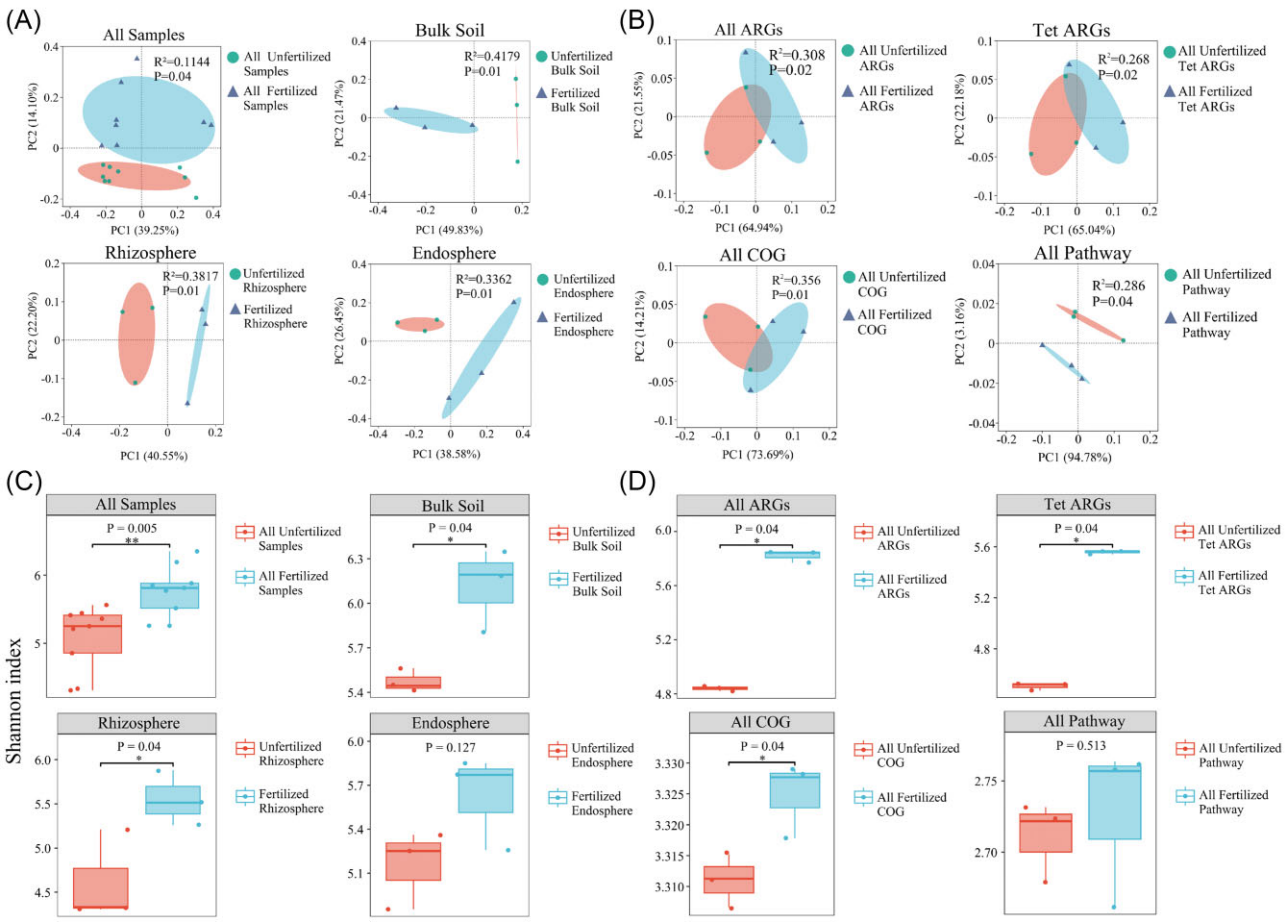
Beta-diversity and alpha-diversity analysis of bacterial communities and functional genes in fertilized and unfertilized samples. PCoA plot showing variation in (A) bacterial communities based on the Bray–Curtis distance and (B) ARGs, Tet ARGs, COG genes, and KEGG pathway functional genes in fertilized and unfertilized rhizosphere soil samples (the significance of differences was evaluated using PERMANOVA). The *x*-axis and *y*-axis represent the two selected principal axes, and the percentage represents the interpretation value of the principal axis for the difference in sample composition; the scale of *x*-axis and *y*-axis is a relative distance, which has no practical significance. (C) Alpha diversity (based on the Shannon index) of the bacterial community in all fertilized and unfertilized samples, and (D) alpha diversity (based on the Shannon index) of ARGs, Tet ARGs, COG genes, and KEGG pathway functional genes in fertilized and unfertilized rhizosphere soil samples. Each point represents different samples with and without fertilization, and the value of *y*-axis represents the Shannon index value of each sample. Kruskal–Wallis nonparametric test was used to obtain the *P*-value of the difference between groups, and Dunn’s test was used to test the significance of the difference. * indicates significant differences at *P* < .05; ** indicates highly significant differences at *P* < .01.

Unconstrained PCoA revealed significant differences in beta diversity between fertilized and unfertilized samples. Significant differences were observed in the composition of the bacterial community in fertilized and unfertilized bulk soil, rhizosphere soil, and root endosphere (Fig. 1A). Significant differences were observed in the beta-diversity of ARGs, Tet ARGs, COG, and KEGG pathway functional genes in fertilized and unfertilized rhizosphere soil samples (*P* < .05) (Fig. 1B). A significant difference in the alpha diversity of bacterial communities based on the Shannon index was observed between fertilized and unfertilized samples. The alpha diversity of bacterial communities was significantly higher in fertilized samples in bulk soil, rhizosphere soil, and root endosphere than in unfertilized samples (Fig. 1C). The alpha diversity of ARGs, Tet ARGs, and COG functional genes was significantly higher in rhizosphere soil in fertilized samples than in unfertilized samples; no significant differences were observed in the alpha diversity of KEGG pathway functional genes between fertilized and unfertilized samples (Fig. 1D).

### Significant enrichment and depletion of bacterial OTUs and ARGs

**Figure 2.**
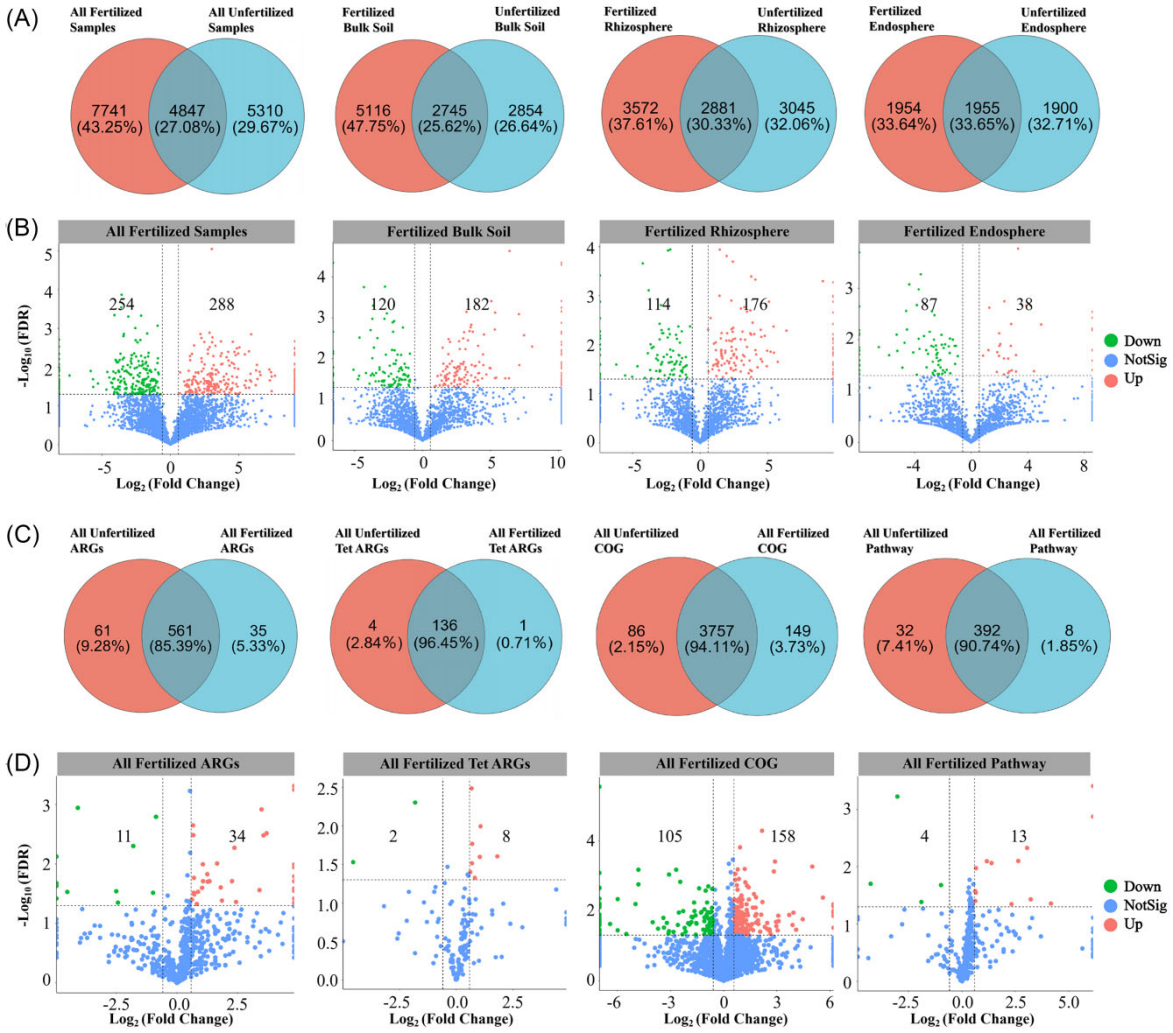
Analysis of the differentially expressed genes of bacterial OTUs and ARGs in fertilized and unfertilized samples. (A) Differences in the number of bacterial OTUs between all fertilized and unfertilized samples, bulk soil, rhizosphere soil, and root endosphere. (B) Enriched and depleted bacterial OTUs in fertilized bulk soil, rhizosphere soil, and root endosphere using unfertilized bulk soil, rhizosphere soil, and root endosphere as controls, respectively. Each point represents an individual OTU, and the position along the *x*-axis represents the abundance fold change compared to unfertilized bulk soil, rhizosphere soil, and root endosphere. The *y*-axis is −Log 10 (FDR) obtained by correcting the *P*-value of the significant difference. The closer the point is to the top of the graph, the more significant the difference is. (C) Differences in the number of all ARGs, Tet ARGs, COG genes, and KEGG pathway genes. (D) Enriched and depleted ARGs, Tet ARGs, COG genes, and KEGG pathway genes in fertilized samples compared with ARGs, Tet ARGs, COG genes, and KEGG pathway genes in unfertilized samples, respectively.

The number of unique OTUs in fertilized samples was as high as 7741, and the number of OTUs was significantly higher in fertilized samples than in unfertilized samples. The number of unique OTUs was also significantly higher in fertilized bulk soil and rhizosphere soil samples than in unfertilized bulk soil and rhizosphere soil samples. No significant difference was observed in the composition of OTUs between fertilized and unfertilized root endosphere samples. Differential expression analysis based on OTU revealed that 288 OTUs were significantly enriched in fertilized samples when the bacterial OTUs of the unfertilized samples were used as a control, and this was significantly higher than the number of depleted OTUs. The number of enriched OTUs was higher in the fertilized bulk soil and rhizosphere soil than the number of depleted OTUs when the OTUs in bulk soil, rhizosphere soil, and root endosphere were used as controls; only 38 OTUs were enriched in the root endosphere, and this was significantly lower than the number of depleted OTUs (Fig. 2A and B).

A total of 657 ARGs and 142 Tet ARGs were detected at the drug class level via the CARD. The number of shared ARGs, Tet ARGs, COG genes, and KEGG pathway genes the fertilized and unfertilized rhizosphere soil samples was higher than the number of unique genes, and the number of shared ARGs and Tet ARGs was 561 and 136, respectively. The number of ARGs, Tet ARGs, COG genes, and KEGG pathway enriched genes in fertilized samples was significantly higher than the number of depleted genes when the numbers of ARGs, Tet ARGs, COG genes, and KEGG pathway genes in unfertilized samples were used as controls (Fig. 2C and D).

**Figure 3.**
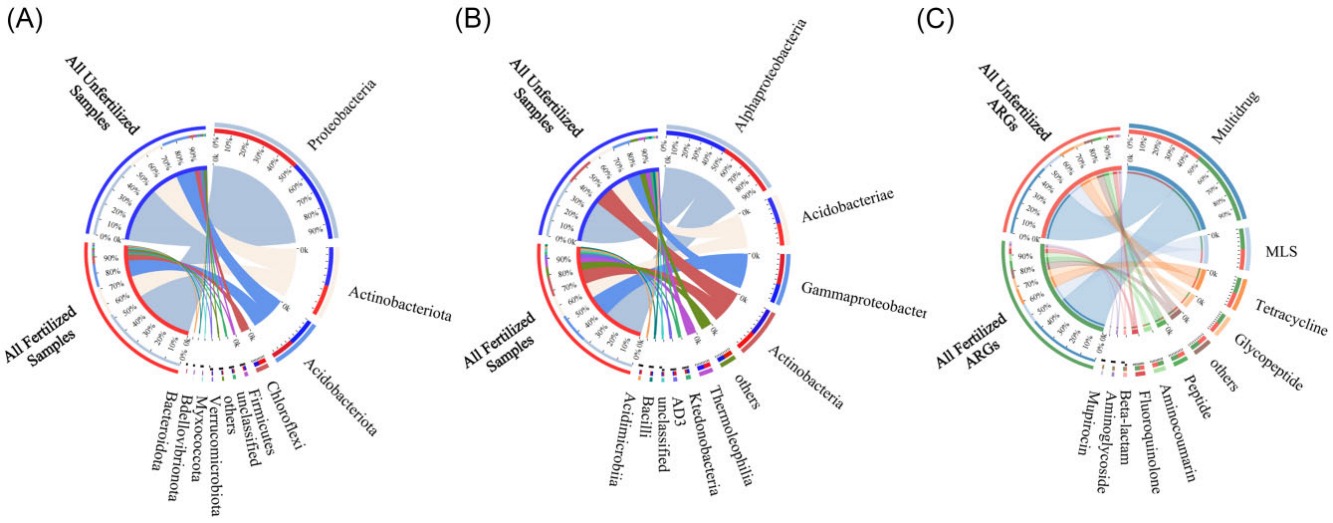
Relative abundances of the top 10 differential bacterial (A) phyla and (B) classes in fertilized and unfertilized samples; (C) relative abundances of the top 10 differential ARGs of different antibiotic classes in fertilized and unfertilized rhizosphere soil samples.

The most abundant bacterial phyla were Proteobacteria, Actinobacteriota, and Acidobacteriota. The relative abundance of Proteobacteria was as high as 53.65% and 49.05% in fertilized and unfertilized samples, respectively. The most abundant bacterial class was Alphaproteobacteria, which accounted for 34.68% of the fertilized samples and 37.11% of the unfertilized samples. The abundances of Acidobacteriae, Gammaproteobacteria, and Actinobacteria were also relatively high (Fig. 3A and B; Tables S4 and S5, Supporting Information). The analysis of the top 10 ARGs at the antibiotic class level showed that the relative abundance of multidrug in fertilized and unfertilized rhizosphere soil samples was as high as 45.21% and 45.60%, respectively, and the abundances of MLS (macrolide–lincosamide–streptogramins) and Tet were also relatively high (Table S6, Supporting Information; Fig. 3C).

Significant differences were observed in bacterial phyla and classes between fertilized and unfertilized samples. Significant differences were observed in the relative abundance of the three dominant bacterial phyla between the fertilized and unfertilized samples. Significant differences were observed in eight dominant bacterial classes between fertilized and unfertilized samples, and the most pronounced difference between fertilized and unfertilized samples was observed for Actinobacteria (Figures S1 and S2, Supporting Information). Significant differences were observed in the relative abundances of all ARGs between fertilized and unfertilized rhizosphere soil samples. The abundances of the 10 most differentially abundant ARGs were significantly higher in fertilized samples than in unfertilized samples (Figure S3, Supporting Information).

### Effects of environmental factors on bacterial communities and ARGs

**Figure 4.**
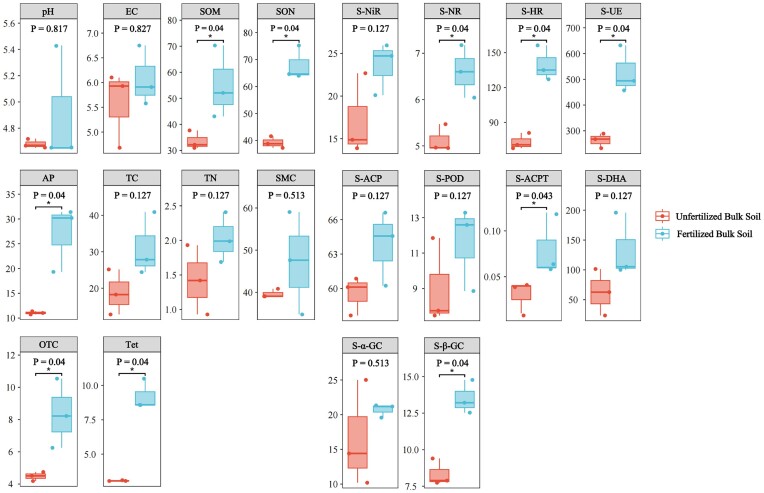
Soil environmental factors in fertilized and unfertilized sample plots of *C. camphora* forests. * indicates significant differences (*P* < .05). Soil physical and chemical properties include pH, EC, SOM, SON, AP, TC, TN, and SMC. Soil enzymes include S-NiR, S-NR, S-HR, S-UE, S-ACP, S-POD, S-ACPT, S-DHA, S-α-GC, and S-β-GC. Each point represents bulk soil with fertilization and without fertilization, and the *y*-axis value represents the values of soil physical and chemical properties and enzyme activity of each sample. Kruskal–Wallis nonparametric test was used to obtain the *P*-value of the difference between groups, and Dunn’s test was used to test the significance of the difference.

**Figure 5.**
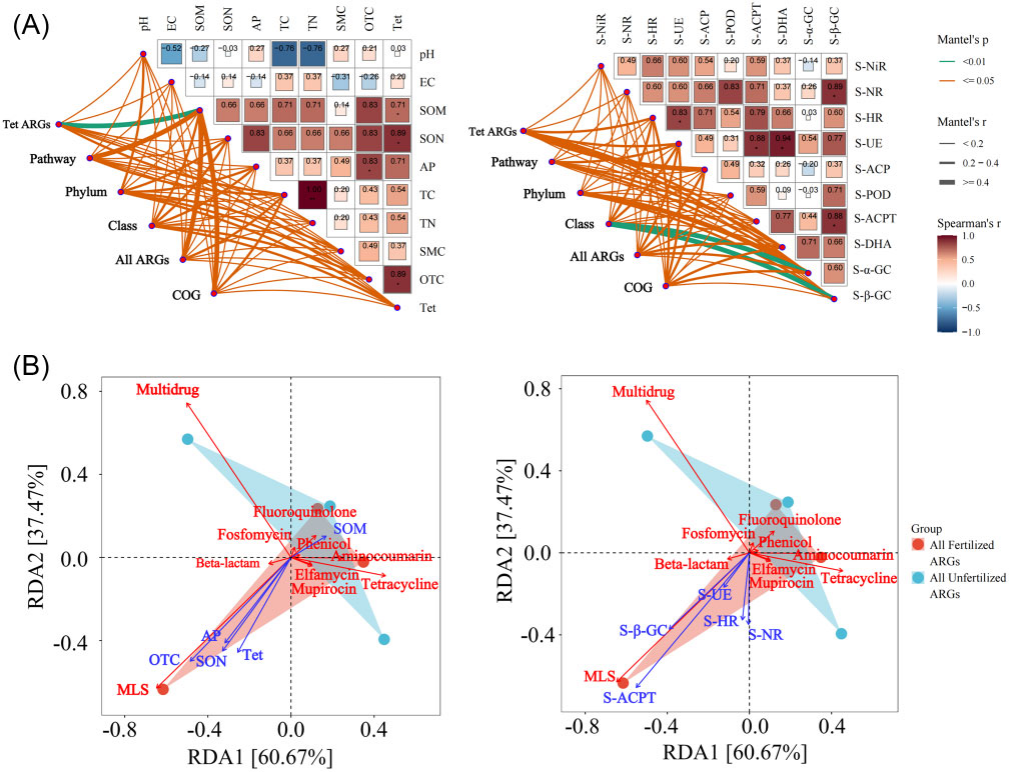
Correlation analysis of functional genes, bacterial communities, and environmental factors. Fertilized and unfertilized rhizosphere soil samples. (A) Functional gene composition and correlation heatmaps of bacterial phyla and classes and environmental factors. Correlations between different environmental factors were represented by Spearman correlation coefficients. The size and color depth in the boxes indicate the correlations. Dunn’s test was used to test the significance of the difference. * indicates that the difference is significant (*P* < .05), and ** indicates that the difference is highly significant (*P* < .01). The thickness of the line indicates the strength of the correlation inferred by the Mantel tests, and the different colors indicate significant differences. (B) RDA based on the Bray–Curtis distance was used to characterize the relationship of ARGs with soil physicochemical factors, OTC, Tet, and soil enzyme activity. The *x*-axis and *y*-axis represent the two selected principal axes, and the percentage represents the interpretation value of the principal axis for the correlation of samples; the scale of *x*-axis and *y*-axis is the relative distance, which has no practical significance. The arrows indicate the lengths and angles between explanatory and response variables and reflect their correlations. Different samples are marked with different colors.

Following the application of chicken manure organic fertilizer, the ground diameter, tree height, crown width, and total biomass of *C. camphora* forests significantly increased (Table S1, Supporting Information). To determine the effects of applying chicken manure organic fertilizer on environmental factors, we measured the pH, EC, SOM, SON, AP, TC, TN, and SMC of fertilized and unfertilized samples from *C. camphora* forests. We also measured the content of various soil enzymes, including S-NiR, S-NR, S-HR, S-UE, S-ACP, S-POD, S-ACPT, S-DHA, S-α-GC, and S-β-GC. In addition, the content of OTC and Tet was determined. The application of chicken manure organic fertilizer significantly increased the content of SOM, SON, AP, OTC, Tet, S-NR, S-HR, S-UE, S-ACPT, and S-β-GC, and no significant differences were observed in the pH, EC, TC, TN, SMC, S-NiR, S-ACP, S-POD, S-DHA, and S-α-GC between fertilized and unfertilized samples (Fig. 4). Mantel tests were conducted on the functional genes, bacterial phyla and classes, and environmental factors. Environmental factors were significantly correlated with all ARGs, Tet ARGs, COG genes, KEGG pathway genes, and bacterial phyla and classes (Fig. 5A).

An RDA of ARGs at the antibiotic class level was conducted on environmental factors showing significant differences between fertilized and unfertilized samples. In the RDA, these environmental factors explained 98.04% of the variation in the composition of ARGs. MLS ARGs were the dominant ARGs; they were significantly positively correlated with SON, AP, OTC, Tet, S-NR, S-HR, S-UE, S-ACPT, and S-β-GC and significantly negatively correlated with SOM. By contrast, fluoroquinolone, aminocoumarin, and Tet ARGs were significantly positively correlated with SOM (Fig. 5B). According to the RDA analysis, these environmental factors explained 97.99% and 92.13% of the variation in the composition of bacterial phyla and classes, respectively. The abundance of Acidobacteria was significantly positively correlated with SON, AP, OTC, Tet, S-NR, S-HR, S-UE, S-ACPT, and S-β-GC, and the abundance of Proteobacteria was significantly positively correlated with SOM (Figure S4a, Supporting Information). The abundance of Actinomycetia was significantly negatively correlated with SON, SOM, AP, OTC, Tet, S-NR, S-HR, S-UE, S-ACPT, and S-β-GC (Figure S4b, Supporting Information).

### Effects of environmental factors on network-level features

**Figure 6.**
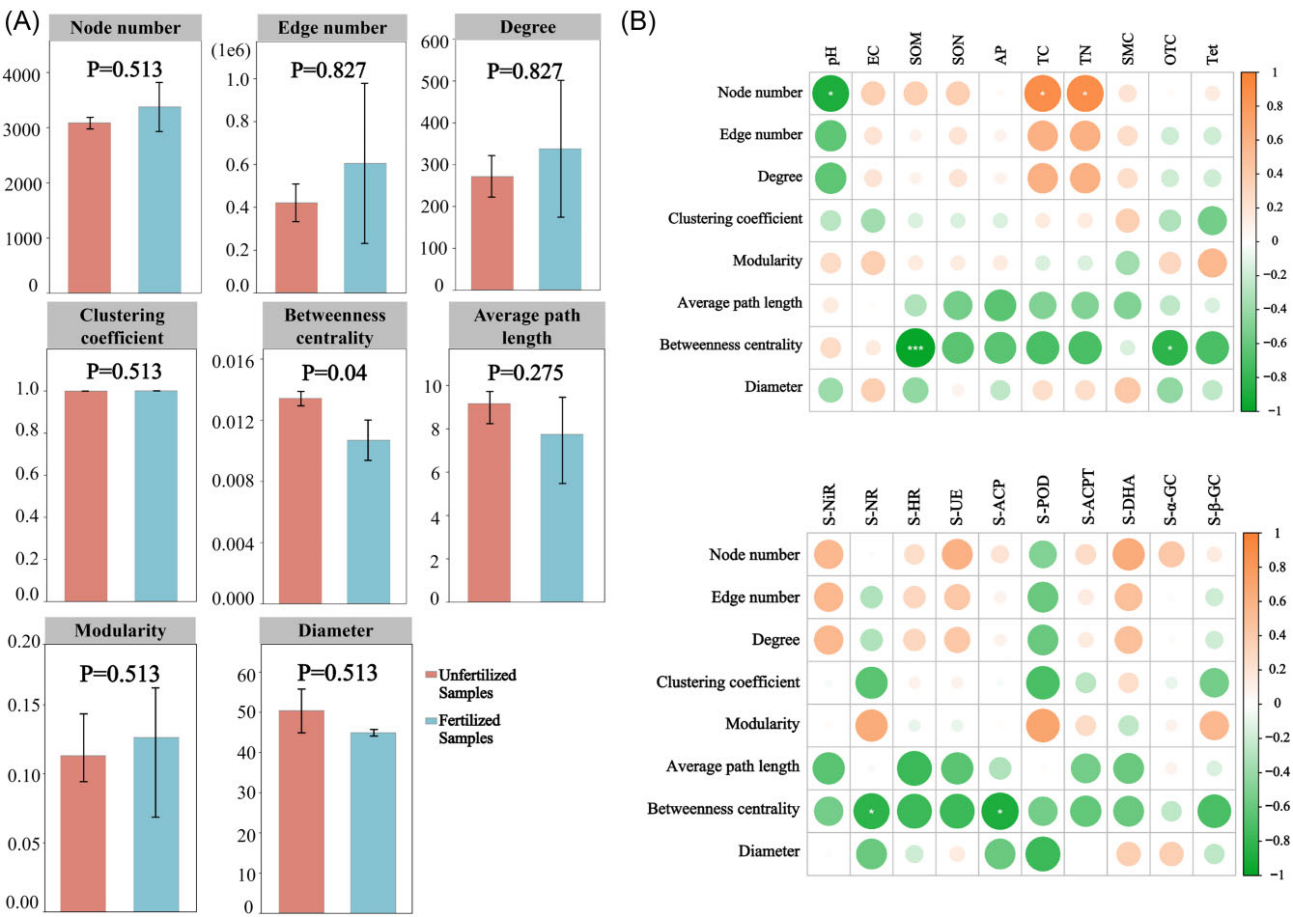
Correlation analysis between environmental factors and network-level topological features. In both fertilized and unfertilized samples, (A) a Wilcoxon rank-sum test was used to compare the network-level topological features of bacterial communities to evaluate the significance of differences between fertilized and unfertilized samples; (B) the correlations between environmental factors and network-level topological features were calculated based on MRMs, and the size and color depth of bubbles in the squares indicate the magnitude of the correlation (positive *R*^2^ values indicate positive correlations, and negative values indicated negative correlations). Wilcoxon rank-sum test was used to test the significance of the difference. * indicates significant differences (*P* < .05), and *** indicates highly significant differences (*P* < .001). The *y*-axis value represents the value of each network-level topological features.

We calculated the network-level topological features of the bacterial community in fertilized and unfertilized samples. Significant differences were observed in the betweenness centrality values in the fertilized and unfertilized samples, and no significant differences were observed in the number of nodes, number of edges, degree, clustering coefficient, modularity, average path length, and diameter (Fig. 6A). We determined the correlations between environmental factors and network-level topological features. The number of nodes was significantly negatively correlated with pH and significantly positively correlated with TC and TN. Betweenness centrality was significantly negatively correlated with SOM, OTC, S-NR, and S-ACP (Fig. 6B). However, no significant differences were observed in the number of edges, degree, clustering coefficient, modularity, average path length, and diameter.

## Discussion

### Composition and diversity of the bacterial communities and ARGs in *C. camphora* forests

Following the application of chicken manure organic fertilizer, all the growth indexes of *C. camphora* forests significantly increased, which suggested that fertilizer application played a key role in promoting the growth and development of *C. camphora* forests. Fertilization can also lead to changes in the soil microbial community. Microbial communities play key roles in maintaining the health of agroforestry ecosystems by mediating the formation of soil and biochemical processes, including residue decomposition and nutrient cycling (Qiu et al. 2014). Fertilization is an important management practice that has a major effect on the structure of soil microbial communities; consequently, fertilizer application can lead to major changes in the community composition and diversity of soil microorganisms (Allison et al. 2008, Hartman et al. 2015, Su et al. 2015). Previous studies have shown that long-term fertilization can lead to substantial increases in the diversity of ARGs in soil under long-term organic fertilizer application (Xie et al. 2018b, Kang et al. 2022). This finding is consistent with the significant differences in the composition and structure of the bacterial community and ARGs between fertilized and unfertilized samples observed in our study; the alpha diversity of the soil bacterial community and ARGs significantly increased following chicken manure organic fertilizer. The retention of some antibiotics in feces might selectively favor the evolution of ARGs in soil (Xie et al. 2018a). Most of the antibiotics applied are excreted in feces and eventually transferred to the soil (Binh et al. 2008, Zhu et al. 2013, Jechalke et al. 2014, Tang et al. 2015). In our study, the content of OTC and Tet in chicken manure organic fertilizer and unfertilized soil was determined. The application of chicken manure organic fertilizer led to significant increases in the content of OTC and Tet antibiotics. Therefore, the increase in the content of Tets antibiotics following fertilization might drive significant increases in the diversity of ARGs.

Analysis of the composition of OTUs in different sample types revealed that the number of unique OTUs was significantly higher in bulk soil and rhizosphere soil than in root endosphere in both fertilized and unfertilized treatments. The number of significantly enriched OTUs in fertilized root endosphere was the lowest compared with that in unfertilized root endosphere. This finding suggests that the selection imposed by the roots of plants is unique to that imposed by other plant organs. Most functional genes and ARGs are common in fertilized and unfertilized rhizosphere soil samples; thus, the application of chicken manure might have little effect on the diversity of functional genes. In addition, the main bacterial phyla detected in this study include Proteobacteria, Actinobacteriota, and Acidobacterota. In the fertilized and unfertilized samples, the relative abundance of Proteobacteria accounted for 53.65% and 49.05% of all bacteria, respectively. The most abundant bacterial classes were Alphaprotobacteria, Acidobacteria, Gammaprotebacteria, and Actinobacteria. These patterns are consistent with the results of a previous study (Chen et al. 2022a) that observed the same dominant bacterial species in *C. camphora*.

We found that the relative abundance of ARGs was significantly higher in fertilized samples than in unfertilized samples. This finding is consistent with the results of a previous study (Chen et al. 2016) showing that the abundance of ARGs in farmland soil significantly increased under the long-term application of chicken manure fertilizer. Animal manure mediates the transport of antibiotics and ARGs into the soil environment, which makes the soil a reservoir of antibiotics and ARGs (Li et al. 2015a). Thus, the abundances of ARGs in the soil increase following the application of chicken manure organic fertilizer. However, significant increases in the abundances of ARGs also lead to increases in environmental pollution risk, and appropriate management practices need to be implemented to mitigate these risks. For example, high-temperature composting can kill bacterial hosts and reduce the number of ARGs in feces, and the transferability and reproducibility of genes preclude the complete elimination of ARGs (Wang et al. 2015, Xie et al. 2016). Thus, the use of antibiotics should be reduced to mitigate increases in the abundances of ARGs in the environment (Vikesland et al. 2017, Xie et al. 2018a).

### Relationships of environmental factors with bacterial communities and ARGs

Soil physical and chemical properties and soil enzyme activities are important properties of the soil environment that play a key role in the transformation of nutrients and the decomposition of OM (Qi et al. 2016). The application of chicken manure organic fertilizer significantly increased the content of SOM, SON, AP, S-NR, S-HR, S-UE, S-ACPT, and S-β-GC. Previous studies examining the effects of manure fertilizer application have shown that animal manure helps increase biological activity and soil fertility, improve soil physical and chemical properties (Adebola et al. 2017, Onunwa et al. 2021), and promote the release of soil nutrients and the activity of soil enzymes (Adubasim et al. 2018, Ogumba et al. 2020).

Several properties of soil affect the enrichment, migration, and transformation of ARGs, and the physical and chemical properties and enzyme activities in soil have a significant effect on the dissemination and distribution of ARGs in the environment (You et al. 2018, Qiu et al. 2021). According to an RDA of environmental factors and ARGs, these selected environmental factors explained a total of 98.04% of the variation in the composition of ARGs; environmental factors had a significant effect on the abundance of ARGs. The results of this study showed that MLS ARGs, which were the dominant ARGs, were significantly positively correlated with various physical and chemical factors and soil enzyme activities, and SON, AP, S-NR, S-HR, S-UE, S-ACPT, and S-β-GC might promote increases in the abundances of MLS ARGs. SOM plays a key role in mediating the spread of ARGs (Li et al. 2021). Increases in the content of SOM might also cause increases in the abundances of fluoroquinolone, aminocoumarin, and Tet. MLS ARGs were also significantly positively correlated with the content of OTC and Tet, and significant increases in the OTC and Tet content following fertilization might increase the abundances of MLS ARGs to a certain extent. Significant increases in the content of SOM, SON, AP, S-NR, S-HR, S-UE, S-ACPT, and S-β-GC following the long-term application of chicken manure organic fertilizer partially explained increases in the abundance of total ARGs. Consistent with the results of Xu et al. (2022), we found that environmental factors might be the most important for driving changes in the abundance and distribution of ARGs following the application of chicken manure organic fertilizer. Therefore, we speculate that environmental factors might have the strongest effects on interactions between microorganisms and ARGs.

An RDA of environmental factors and bacterial communities revealed that the phylum and class levels explained 97.99% and 92.13% of the variation in bacterial community composition, respectively. The dominant bacterial phylum Acidobacteria played key roles in various biogeochemical cycles; Acidobacteria are metabolically diverse and significantly positively correlated with most of the measured environmental factors. OM had a significant effect on soil bacterial diversity (Li et al. 2021), Proteobacteria play key roles in the decomposition and recycling of OM (Dang and Lovell 2016), which might promote increases in the SOM content. Actinomycetia was significantly negatively correlated with all measured environmental factors. The selected environmental factors all had significant effects on the dominant bacterial communities. According to the results of previous studies, the application of organic fertilizers can alter the diversity and composition of bacterial communities by altering the relevant environmental factors of the soil (Chen et al. 2016, 2021).

### Changes in the network-level topology of bacterial communities

We analyzed the network-level topological characteristics of bacterial communities in fertilized and unfertilized samples. The betweenness centrality (i.e. the number of shortest paths passing through a node) was significantly lower in fertilized samples than in unfertilized samples, and no significant differences were observed in the eigenvalues for other topological characteristics. The network-level topology provides insights into interactions among microbial species (Deng et al. 2012). The degree value (i.e. the number of adjacent edges) indicates the number of direct interactions between connected OTUs. Betweenness centrality indicates the importance of interactions between OTUs, and lower betweenness centrality values indicate that microbes are located further away from the core position in the network. These species might have relatively small effects on the interactions between other species in the community (Greenblum et al. 2012, Ma et al. 2016). The results of this study indicate that differences in the interactions between microorganisms in fertilized and unfertilized samples were low. We also conducted MRM analysis to identify environmental factors that could explain network-level topological changes in bacterial communities between fertilized and unfertilized samples. No significant correlations between environmental factors and topological features were observed, with the exception of the number of nodes and betweenness centrality. Most of the measured environmental factors had no significant effect on the topological features of the bacterial community. Changes in network-level topological features of the bacterial community were analyzed in our study; however, the inferred interactions are based on associations between two variables, and these correlations are not sufficient for demonstrating direct interactions between microorganisms and might not reflect actual correlations between taxonomic groups. Additional experimental work is needed to verify whether the associations between microorganisms in our study reflect direct interactions.

## Conclusions

We used 16S rRNA and metagenomic high-throughput sequencing technology to characterize the effects of chicken manure organic fertilizer on the composition and diversity of bacterial communities and ARGs in the rhizosphere soil of *C. camphora* forests. We also studied the relationships of environmental factors with bacterial communities and ARGs. Significant differences in the composition of bacterial communities and ARGs were observed between fertilized and unfertilized rhizosphere soil. The abundance and diversity of rhizosphere soil bacterial communities and ARGs significantly increased following the application of chicken manure organic fertilizer. The changes of soil physicochemical factors, soil enzyme activity, and Tet antibiotic content have significant effects on bacterial community and ARGs. Tets antibiotics and environmental factors were the main factors affecting changes in the abundance and diversity of rhizosphere soil ARGs following the application of chicken manure organic fertilizer.

## Supplementary Material

fiad155_Supplemental_Files
