# Supplementary figures and images for: Chicken manure application alters microbial community structure and the distribution of antibiotic-resistance genes in rhizosphere soil of Cinnamomum camphora forests
Source: FEMS Microbiol Ecol. 2023 Nov 24;99(12):fiad155. doi: 10.1093/femsec/fiad155 (PMC10710299; doi:10.1093/femsec/fiad155)

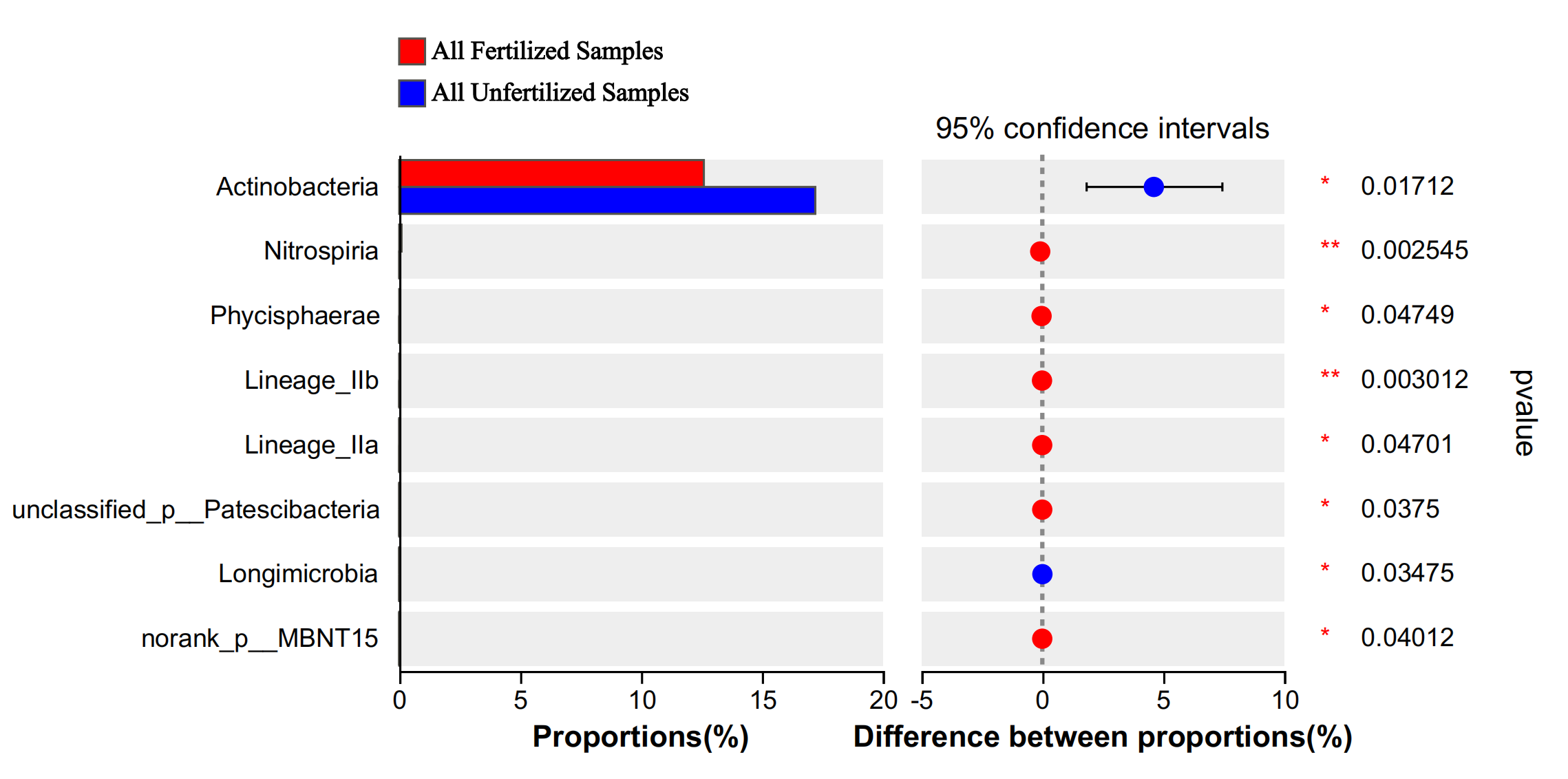

Supplement: fiad155_Supplemental_Files [file fiad155_supplemental_files.zip › Supplementary_data Fig S2.png]

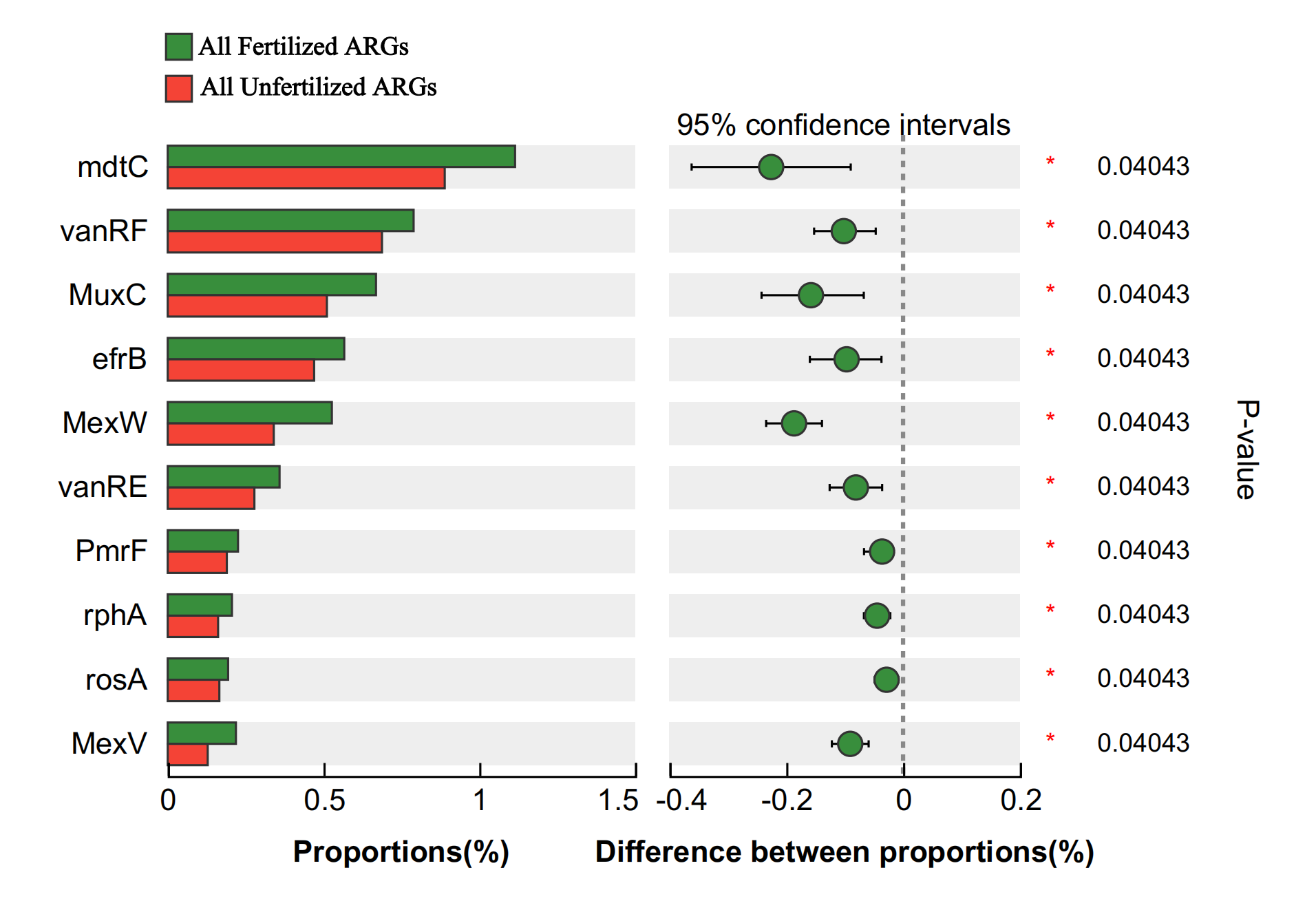

Supplement: fiad155_Supplemental_Files [file fiad155_supplemental_files.zip › Supplementary_data Fig S3.png]

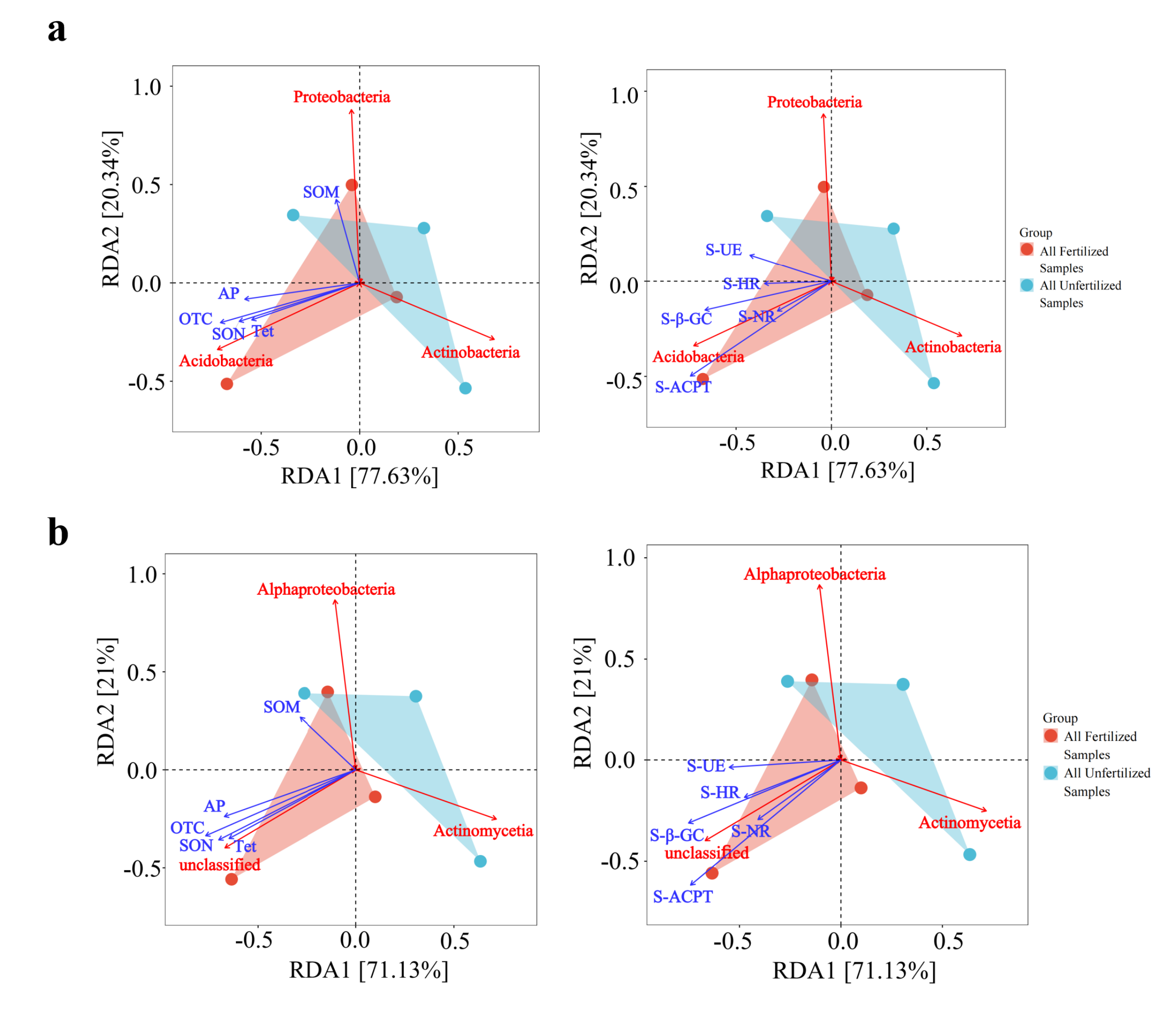

Supplement: fiad155_Supplemental_Files [file fiad155_supplemental_files.zip › Supplementary_data Fig S4.png]

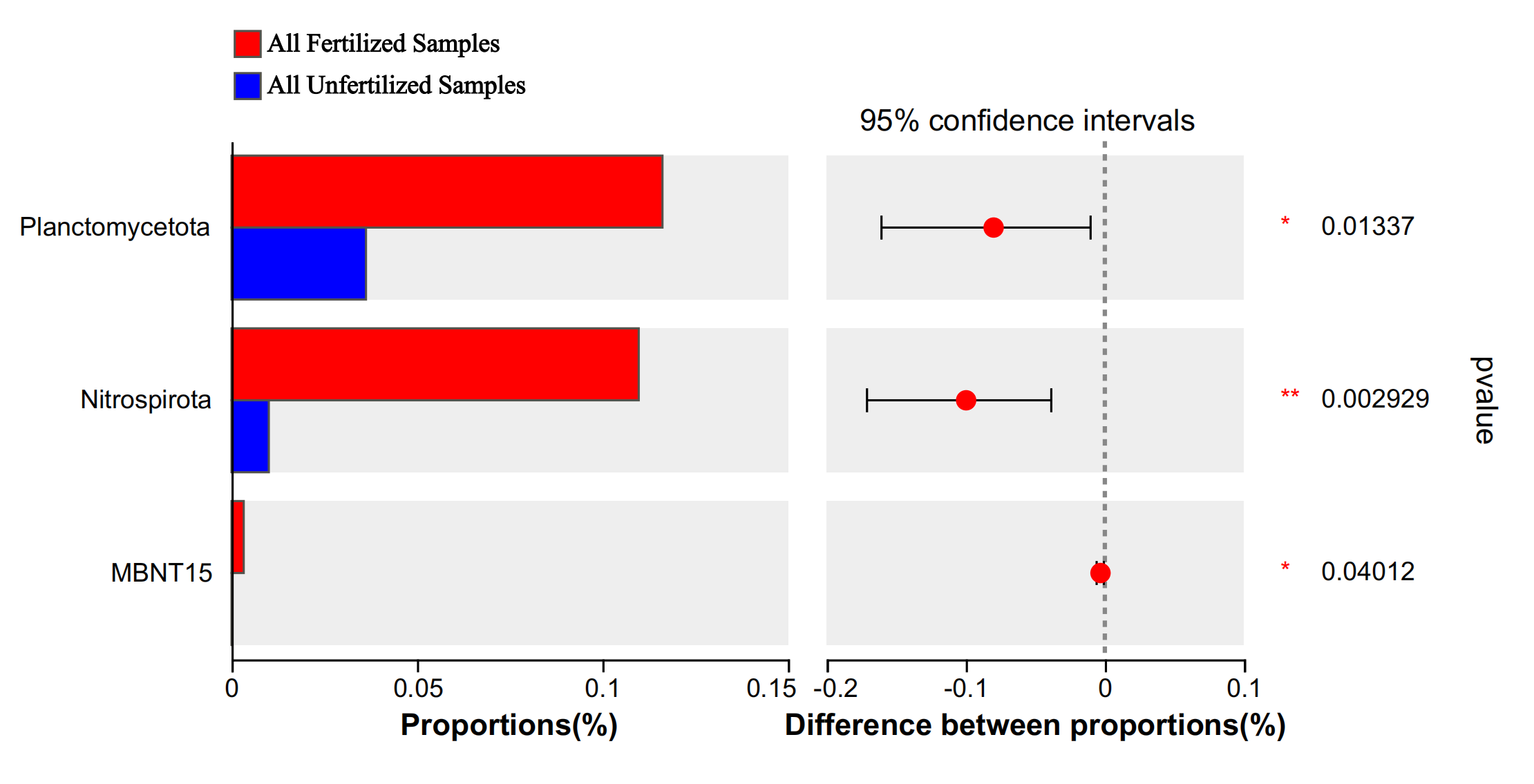

Supplement: fiad155_Supplemental_Files [file fiad155_supplemental_files.zip › Supplementary_data Fig S1.png]
